# The Patient Perspectives on Future Therapeutic Options in NASH and Patient Needs

**DOI:** 10.3389/fmed.2019.00061

**Published:** 2019-04-03

**Authors:** Nigel Cook, Andreas Geier, Andreas Schmid, Gideon Hirschfield, Achim Kautz, Jörn M. Schattenberg, Maria-Magdalena Balp

**Affiliations:** ^1^Novartis Pharma AG, Basel, Switzerland; ^2^Division of Hepatology, University Hospital Würzburg, Würzburg, Germany; ^3^Health Management, School of Law and Economics, University of Bayreuth, Bayreuth, Germany; ^4^Toronto Centre for Liver Disease, University Health Network, University of Toronto, Toronto, ON, Canada; ^5^Leberhilfe Projekt gUG, Köln, Germany; ^6^Department of Medicine, University Medical Center Mainz, Mainz, Germany

**Keywords:** patient preference, non-alcoholic fatty liver disease (NAFLD), non-alcoholic steatohepatitis (NASH), adaptive choice-based conjoint, liver disease, EQ5D-5L, patient-based evidence, patient-reported outcomes

## Abstract

**Background:** Non-alcoholic steatohepatitis (NASH) is a chronic liver disease with severe complications and without approved therapies. Currently, there is limited data on the overall burden of the disease for patients or on patient needs and preferences. This study investigates patient preferences in relation to potential future therapies for NASH. In addition, the factors that are relevant to patients and their importance in relation to future treatment options are explored.

**Method:** Telephone in-depth interviews (TDIs) preceded an online 30-min quantitative survey. The online survey included (1) multiple choice questions (MCQs) on NASH diagnosis and disease background. (2) An exercise to determine patients' satisfaction levels with information provided at diagnosis, and to explore symptomatology in detail. (3) Exercises to evaluate potential new products and product attributes, including a “drag and drop” ranking exercise, and an adaptive choice-based conjoint exercise (ACBC). (4) The EQ-5D-5L questionnaire and the Visual Analog Scale (VAS), which measures patients' health status. (5) Collection of socio-demographic data, and (6) Questions to measure patient satisfaction with the survey.

**Results:** There were 166 patients included in this study from Canada [*n* = 36], Germany [*n* = 50], the UK [*n* = 30], and USA [*n* = 50]. Fifty seven percent of patients [*n* = 94] had had a liver biopsy for confirmation of NASH. Patients were often unable to link their symptoms to NASH or other conditions. ACBC results showed that efficacy, defined as “impact on liver status” was the single most important attribute of a potential future NASH therapy. Other attributes considered to have secondary importance included impact on weight, symptom control and the presence of side effects. The EQ-5D utility score was 0.81 and VAS = 67.2.

**Conclusion:** “Impact on liver status” is the primary outcome sought. Patients demonstrate a general lack of understanding of their disease and appeared to be unfamiliar with longer-term consequences of NASH. It is necessary to improve patient understanding of NASH and its progressive nature, and there is a need for improving confirmatory diagnosis and monitoring.

## Introduction

Non-alcoholic steatohepatitis (NASH) is the advanced form of non-alcoholic fatty liver disease (NAFLD) histologically characterized by accumulation of fat, inflammation and fibrosis, which can result in cirrhosis and may progress to hepatocellular carcinoma. There is high variability in the reported prevalence for NAFLD and NASH due to differences in the population studied, definition of the disease, regional aspects, and histological classification systems used to diagnose NASH. There is limited data reporting the prevalence of NASH in the general population, however a number of studies have reported the prevalence of NAFLD patients, to be between 21.9% (1) and 37.2% (2). While the prevalence of biopsy confirmed NASH in NAFLD patients is estimated to be between 15.9% (3) and 70.2% (4). Many patients may not have been identified due to a lack of unique characteristics and the requirement of a liver biopsy to confirm diagnosis. NASH is more frequent among patients with type II diabetes mellitus (T2DM) and obesity and the symptoms related to these conditions could be masking the underlying liver condition and related symptoms.

The role of patients in decision-making regarding their disease management has typically been low, however, the patient voice is increasingly being incorporated into the regulatory process, Health Technology Assessment (HTA), and drug development process (5).

Patient preference has been investigated in other disease areas as patients are uniquely placed to inform on realities of living with their condition and the gaps with current treatment options. Previously a range of methodologies have been used in a number of therapy areas to gain these insights (6–9). There is limited published data available on the disease burden of NASH—in particular related to a patients' health-related quality of life (HRQoL). The preferences exhibited by NASH patients in decisions regarding their management have not need extensively studied. Indeed, a literature search performed at the start of this study on MEDLINE, Cochrane, Health Technology Assessment websites and Embase, November 2016, retrieved only six articles (10–15). This area of research is undoubtedly growing, however, there is a perceivable knowledge gap when it comes to quantifying NASH patient HRQoL and preferences for treatment attributes.

Given the large patient population, uncertainties surrounding treatment profiles and in order to enable informed decision-making by patients, the aim of this study was to examine preferences of patients diagnosed with NASH, to include patient view early in the drug development life cycle. Understanding patient views could contribute to a better characterization of the preferences that patients might have for future therapies. Even accounting for the exploratory nature of this study, the results will improve the understanding of the needs of this patient population and provide regulators, HTA and drug developers the required insights to support decision-making.

## Materials and Methods

**Figure 1:**
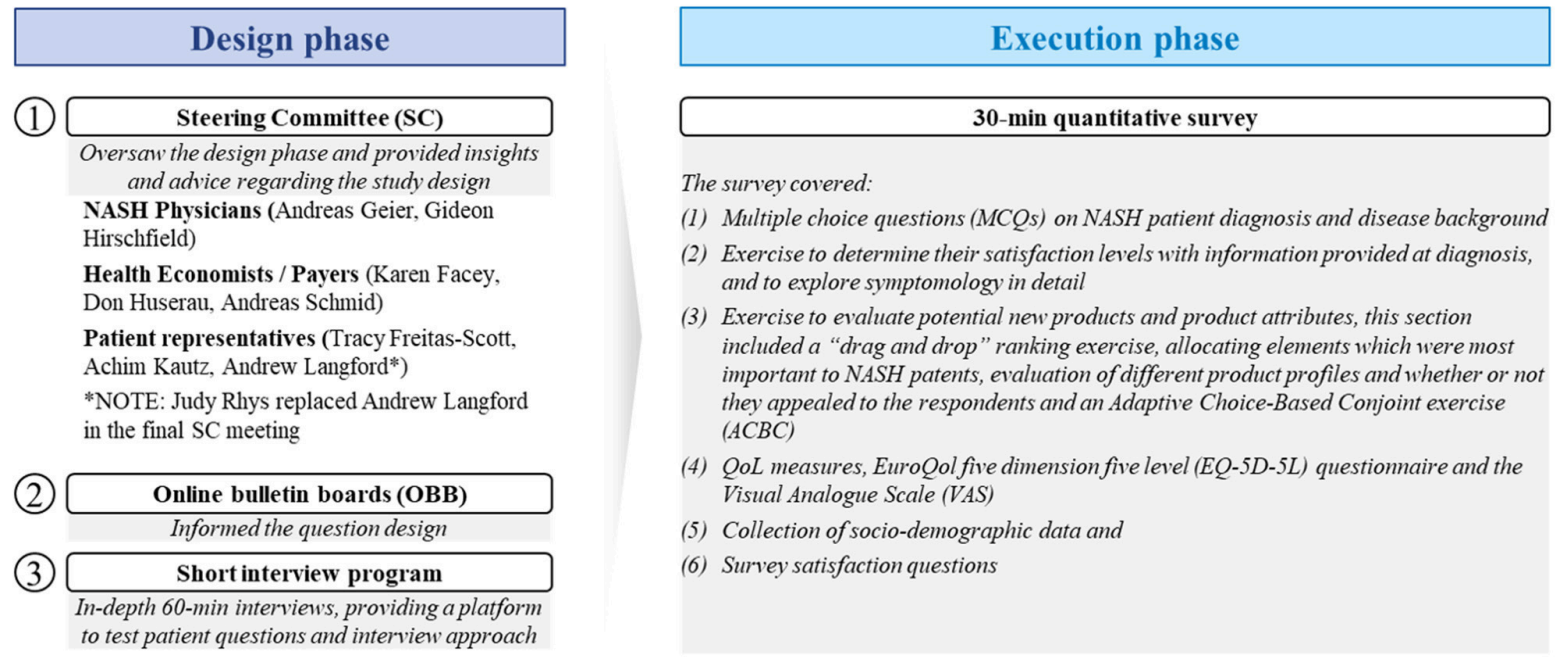
Methodology summary.

The study was conducted in two phases, the design phase and execution phase. To ensure the relevance of the study, several inputs were leveraged during the design phase, which informed the structure of the execution phase (Figure 1).

The aim of the design phase was to ensure a robust methodology for this research. To that end, inputs from patient online bulletin boards (OBB) (16) were considered and an in-depth 60-min telephone interview program with patients was used to test and refine patient survey questions. Interviewers for this phase were experienced in patient research with over 7 years of experience in moderating such discussions. Additionally, a Steering Committee (SC) composed of NASH stakeholders was assembled to oversee the process. The stakeholders included internationally-recognized NASH clinical experts, patient group representatives, a NASH patient, and advisers to HTA/reimbursement bodies (Figure 1).

Insights from the design phase helped shape the materials used in the execution phase, namely the patient screener questions and the questions to be included in the discussion guide. Both the discussion guide and the screener were common to all countries investigated and translated into local languages.

The execution phase included a 30-min on-line self-completed quantitative survey which contained six sections:

1. Multiple choice questions (MCQs) on patients' experiences with diagnosis of NASH and their disease background knowledge.
2. Exercises to determine their satisfaction levels with information provided at diagnosis, and to explore symptomatology in detail.
3. An Adaptive Choice-Based Conjoint exercise (ACBC—an indirect-elicitation method) to measure patients' preferences on benefits and risks, to derive preference weights in a scale that allows for direct comparison and to simulate their choices.
4. EQ-5D-5L questionnaire, a preference-based standardized measure developed by Euroqol (17). It comprises five dimensions: mobility, self-care, usual activities, pain/discomfort, anxiety/depression with a 5-level answer choice ranging from “no problem” to “extreme” problems. A Visual Analog Scale (VAS) on which the patient marks the health status in the day of completion is also included as part of the questionnaire. The EQ-5D-5L utility score can range between 0 (death) and 1 (perfect health), while VAS score ranges from 0 (worst imaginable health) to 100 (best imaginable health).
5. Collection of socio-demographic data.
6. Questions to measure patient satisfaction with the survey.

The recruitment of respondents took place across four countries, Canada, Germany, the United Kingdom (UK), and the United States of America (USA). Patients were recruited for this study via physician referral from various sites (i.e., specialist liver centers, hospitals etc.). The screening criteria required patients to have a confirmed or suspected NASH diagnosis, as determined by either biopsy or transient elastography (FibroScan) or an ultrasound (in the UK only), with a fibrosis stage F2 or F3. All participants were recruited and reimbursed in accordance with the local market research norms and regulations.

The analysis of the results was done qualitatively at the design stage, and discussed with the Steering Committee, and quantitatively after the execution phase, by using Microsoft Excel 2016 Professional Plus to analyse raw quantitative data from the survey, and Sawtooth Software–Lighthouse Studio 9.5.3 and R Studio 1.11.383 to analyse ACBC questions.

The ACBC exercise had three sections, each section being different from the previous but built based upon the answers from the preceding section. The outcome was that each respondent received a customized and personally relevant questionnaire.

- Section A (drag and drop exercise) was used to select the most relevant attributes to feed into the next sections. The patients were asked to rank a pre-defined list of 10 product characteristics from most important to least important to them. Because testing more than 6–7 different attributes in a choice exercise would likely exceed the patients' concentration capabilities and tempt them to apply simple decision heuristics, we decided to limit the number of attributes that will be forwarded to the conjoint part to seven attributes ensuring the quality of the responses. If an attribute was non-considered, we assumed it has zero importance in decision, so all its levels will receive 0 utility.
  The attributes and their discrete levels (the magnitude or category of each attribute) were identified through previous qualitative research and refined by a steering committee (Table 1).
- Section B was used to build a consideration set of hypothetical product profiles and establish any non-compensatory rules (test respondents' sensitivity to changes). The patients were asked to indicate for each hypothetical product profile whether it appeals to them or not. Each hypothetical product profile was made by a profile of 7 relevant attributes (from Section Introduction), each of which represents a level (feature).
- Section C was used to identify the best hypothetical product profile. The patients were asked to choose the most-preferred alternative from a set of considered hypothetical profiles.

**Table 1:** List of attributes and their discrete levels used in ACBC exercise.

**Attributes**	**Levels**
Impact on liver status	My doctor says my liver is unchanged (based on test results)
	My doctor says my liver is better (based on test results)
Impact on weight	No impact on weight
	Weight loss by less than 5% of my current weight
	Weight loss by more than 5% of my current weight
Impact on symptoms possibly linked to your liver disease	No impact on fatigue/tiredness or on stomach pain
	Reduction of fatigue/tiredness
	Reduction of stomach pain
	Reduction of both fatigue/tiredness and stomach pain
Impact on blood sugar (diabetes) and cholesterol	No interaction with any diabetes medication that I am taking
	Lowers the level of LDL (“bad”) cholesterol in my blood
	Makes my diabetes medication less effective (potentially needing to increase its dosing)
Frequency of visits to your doctor(s) for your liver condition	Same amount of visits to my doctor(s) for my liver condition
	More visits to my doctor(s) required for my liver condition
Impact on progression to serious damage to my liver (cirrhosis)	No impact on the progression to serious damage to my liver (cirrhosis)
	Slows down the progression to serious damage to my liver (cirrhosis)
Side-effects: Diarrhea	Causes mild diarrhea (<1 day out of 10)
	Does not cause diarrhea
Side-effects: Nausea	Causes occasional nausea (once a week or less)
	Does not cause nausea
Side-effects: Headache	Causes occasional headache (once a week or less)
	Does not cause headache
Side-effects: Itching	Causes mild to moderate itching
	Does not cause itching

Results were used to determine the part-worth utilities for all the discrete levels of the 10 characteristics (attributes). The results are reported as relative importance scores based on utilities range. Furthermore, the utilities generated from the conjoint analysis are used to estimate the proportion of times that a particular hypothetical product profile (with the attribute levels) will be chosen if the product were to be introduced to the market. This is commonly known as Market Share Simulation.

The study was intended to examine pooled data from all countries and, although country specific results are illustrated, no statistical subgroup analysis was undertaken.

For this study, IRB approval was obtained from Salus IRB for the US, UK, and Canada. While for Germany, the Ethics Committee approval from Rheinland-Pfalz was obtained. These approvals were obtained prior to engaging with patients.

## Results

### The Design Phase

Inputs from the design phase produced a number of findings which were carried forward to the execution phase.

The in-depth 60-min interview program was conducted with a total of 17 patient interviews across Canada [*n* = 6], Germany [*n* = 5], and the UK [*n* = 6]. Of those, 94% [*n* = 16] were classed as obese, while 59% [*n* = 10] were diabetic or pre-diabetic (Table 2). The key findings were similar to those from the OBBs (16). They showed that patients had a poor understanding of NASH and the symptoms associated with it and had trouble differentiating between NASH symptoms and symptoms from other comorbidities. The interviews also showed that the patients frequently receive recommendations from their physician to lose weight and adopt a healthy lifestyle. This advice, however is interpreted by patients as relatively unimportant, as such advice is routinely shared by their physician regardless of the patients' medical condition (e.g., in relation to their comorbid conditions).

**Table 2:** Patient characteristics of patients taking part in the 60-min in-depth qualitative interviews.

	**All countries**	**Canada**	**Germany**	**UK**
Total number of patients	17	6	5	6
Males	8	2	1	5
Females	9	4	4	1
Age range	34–69	34–50	50–69	43–68
Biopsied patients	7	4^*^	2	1
F2	6	3	3	Moderate *n* = 5
F3	5	3	2	Severe *n* = 1
**COMORBIDITIES**				
Obese	16	6	4	6
Diabetic/pre-diabetic	10	3	3	4
Experience depression	5	3	2	0

* *Three patients participating in clinical trials, therefore screened for eligibility for a clinical trial*.

The SC overseeing the research considered these findings and advised that the questions asked to the patients had to have a low cognitive burden and they should include questions which would produce quantifiable answers. The SC also outlined the themes to be probed in the execution phase; namely the diagnosis and disease background, evaluation of efficacy, side-effects, NASH symptoms, full product profiles, and HRQoL. These recommendations were incorporated into the preparation of materials for the online survey.

### Execution Phase

A total of 166 patients (82 female and 84 male) took part in the online 30-min quantitative survey (additional data from the survey can be found in the Supplementary Information Data Files). The mean patient age was 52.02 years. All patients had liver fibrosis stage 2 (F2) [*n* = 106] or stage 3 (F3) [*n* = 60], 94 were confirmed to have NASH via a liver biopsy; 118 respondents were classed as obese in accordance to the national guidelines; and 88 were diabetic or had pre-diabetes (Table 3).

**Table 3:** Overall summary of patient characteristics for the online quantitative survey.

**Country**	**All countries**	**Canada**	**Germany**	**USA**	**UK**
Total number of patients	166	36	50	50	30
Screening criteria	–	NASH diagnosed patients using biopsy/FibroScan			NASH diagnosed patients using biopsy/FibroScan or ultrasound
Diagnosis via biopsy or FibroScan	Biopsy *n* = 94 FibroScan *n* = 87	Biopsy *n* = 12 FibroScan *n* = 24	Biopsy *n* = 45 FibroScan *n* = 21	Biopsy *n* = 25 FibroScan *n* = 34	Biopsy *n* = 12 FibroScan *n* = 8
Number of F2/F3 stage patients	F2 stage *n* = 106 F3 stage *n* = 60	F2 stage *n* = 35 F3 stage *n* = 1	F2 stage *n* = 29 F3 stage *n* = 21	F2 stage *n* = 16 F3 stage *n* = 34	F2 stage *n* = 26 F3 stage *n* = 4
Obesity	114	5	47	44	18
Diabetes or pre-diabetes	88	3	26	45	14
Dyslipidemia	72	1	18	44	9
Hypertension	80	2	24	39	15
Coronary Artery Disease	19	0	5	12	2
Depression	26	5	6	10	5
Sleep apnea	25	1	9	7	8
Joint/bone issues	21	0	2	12	7
Muscle issues	10	4	0	3	3
Gender	Female *n* = 84 Male *n* = 82	Female *n* = 22 Male *n* = 14	Female *n* = 25 Male *n* = 25	Female *n* = 21 Male *n* = 29	Female *n* = 14 Male *n* = 16
Mean age	52.03 years (SD:11.78)	44.75 years (SD: 14.45)	53.5 years (SD:7.76)	53.38 years (SD:11.05)	54.87 years (SD: 11.95)
Employment status (employed full/part time or self-employed)	64.4%	86%	44%	68%	67%
Out of work, student, retired person, homeowner, military or unable to work	27.0%	8%	32%	32%	33%
Preferred not to disclose employment status	8.4%	6%	24%	0%	0%

In most cases (65% of patients [*n* = 108]), patients reported that the first suspicion of having a liver condition came from their family doctor (Table 4). An additional 23% [*n* = 39] of patients first learned of the liver condition upon visiting a hepatologist or a gastroenterologist. Only a minority reported that the first suspicion of having a liver condition was done by a diabetologist (5%, [*n* = 9]) or another healthcare professional (6%, [*n* = 10]).

**Table 4:** Type of physician seen on first suspicion of having a liver condition *(Q: We would now like to cover the medical journey of your liver condition. Please think back to the first visit to your doctor when he or she had a suspicion for the first time that you might have a liver condition. What type of doctor did you see during that particular first visit?)*.

	**TOTAL**	**COUNTRY**			
		**USA**	**Canada**	**Germany**	**UK**
Total	166	50	36	50	30
Family doctor/primary care physician	108	18	35	38	17
Hepatologist/gastroenterologist	39	25	1	9	4
Diabetologist	9	5	0	1	3
Other	10	2	0	2	6

#### Multiple Choice Questions on NASH Patient Diagnosis and Disease Background

**Figure 2:**
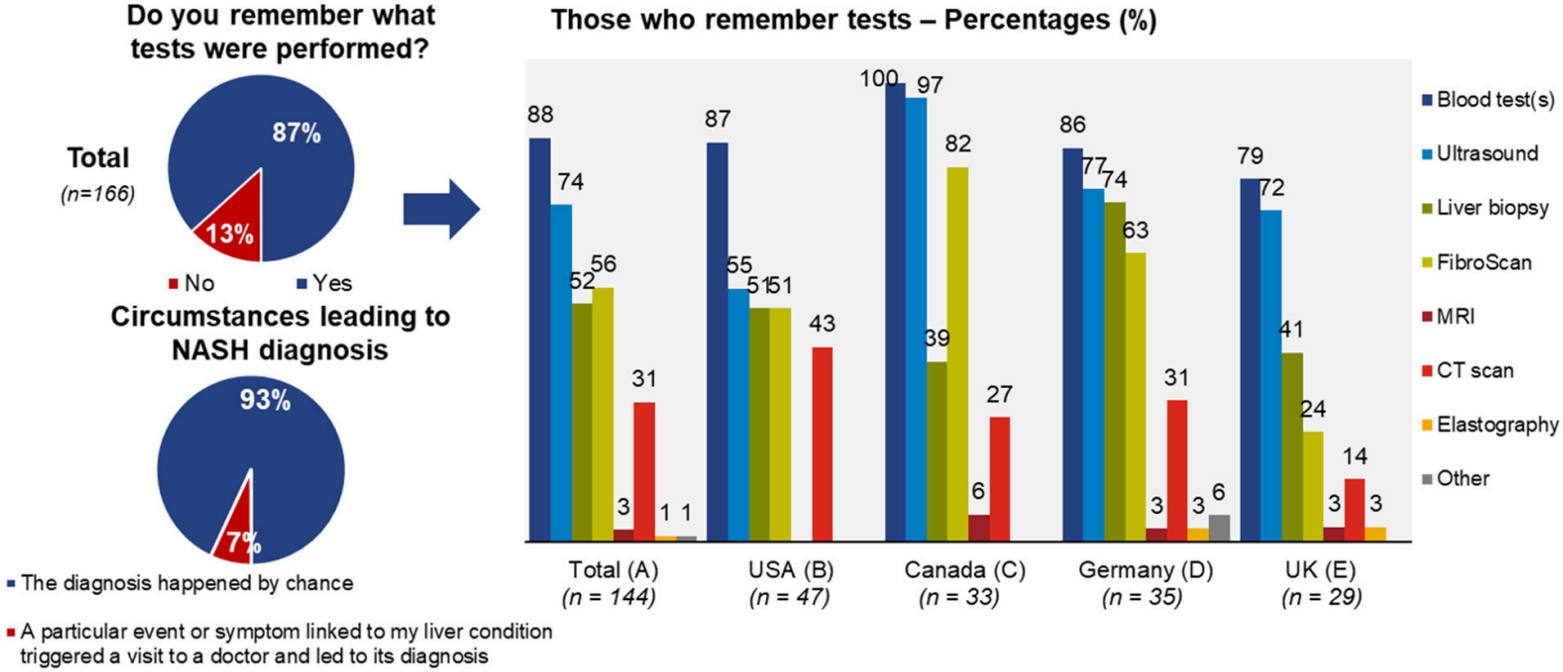
Overview of clinically confirmed or suspected NASH diagnosis across countries.

The first section of the quantitative survey relating to NASH diagnosis and disease background, has shown that 93% of patients across all countries [*n* = 154] believe that the diagnosis happened by chance, and the main tests that patients were able to recall [base = 144 respondents] were the blood tests in 88% of cases [*n* = 127] and ultrasound in 74% of cases [*n* = 106]. Liver biopsy and transient elastography were reported by patients less frequently, in 56% [*n* = 80] and 54% [*n* = 75] of cases, respectively (Figure 2).

#### Information Satisfaction Levels and Symptomatology

**Figure 3:**
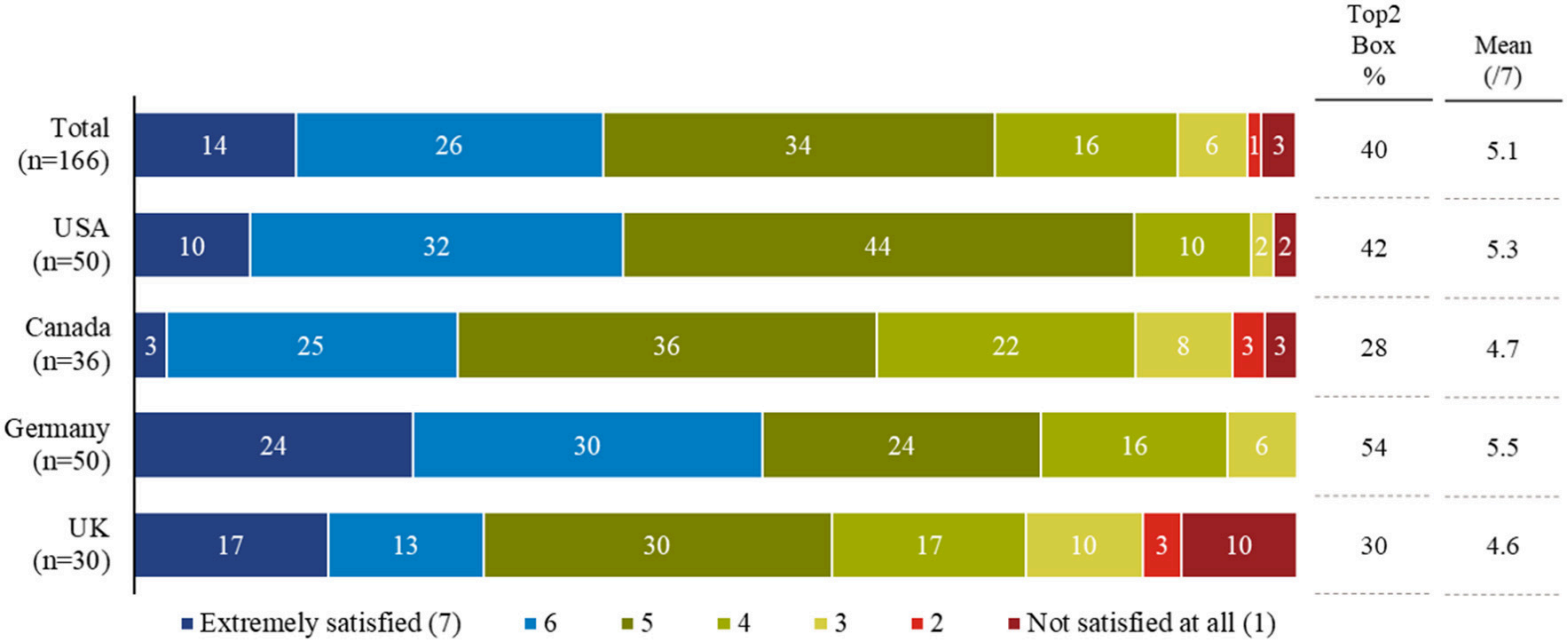
Patient satisfaction with information provided about NASH by their physician on diagnosis.

When patients were asked about their satisfaction with the specific information provided by physicians when NASH was diagnosed, on a 1–7 scale (1 being “not satisfied at all,” and seven being “extremely satisfied”), patients reported a mean satisfaction of 5.1. Those scoring 6 [*n* = 44] or 7 [*n* = 23], together constituted 40% [*n* = 67] of all respondents (Figure 3).

**Figure 4:**
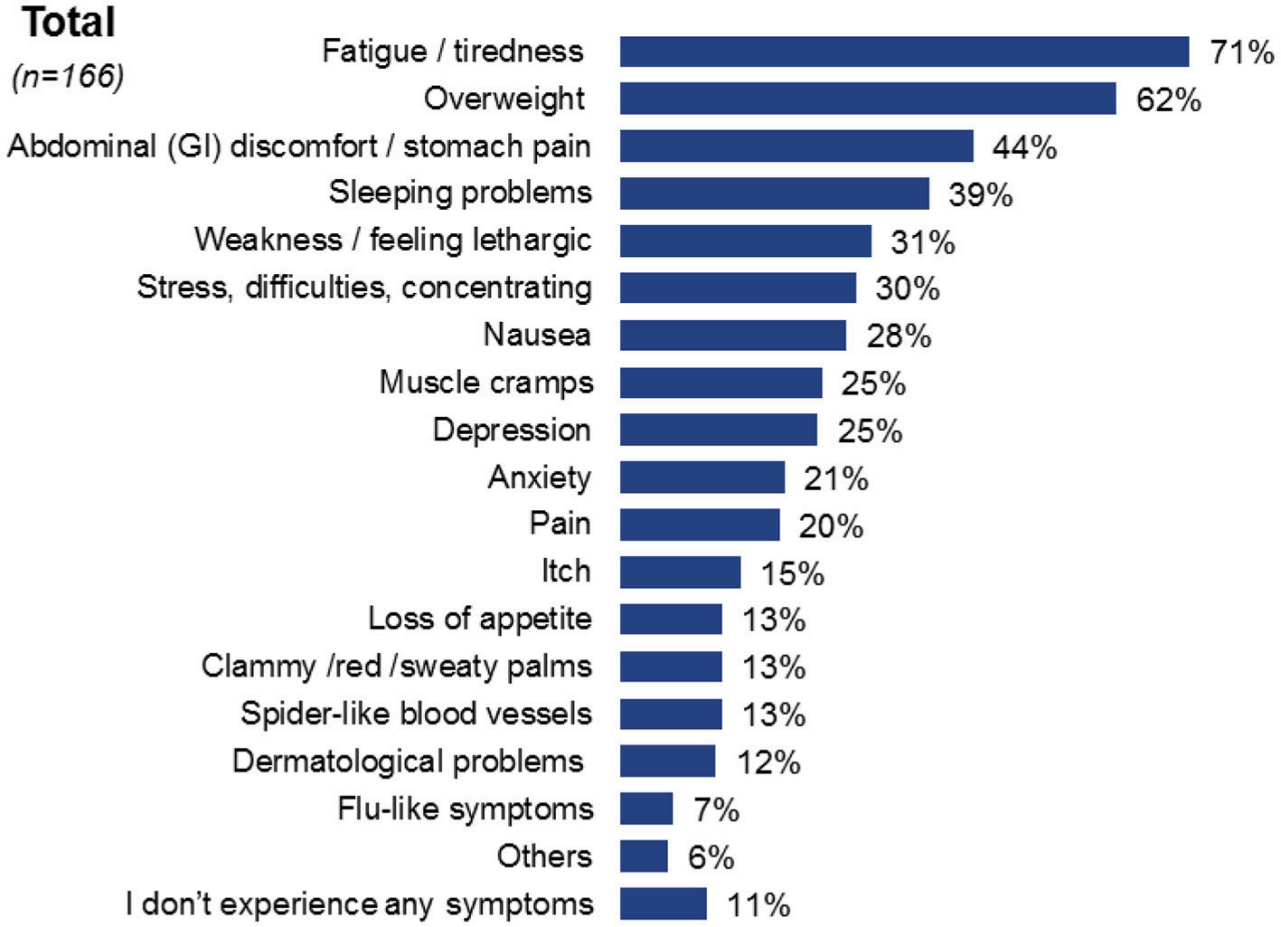
Overall reported symptoms of NASH patients across the four countries of this survey.

**Figure 5:**
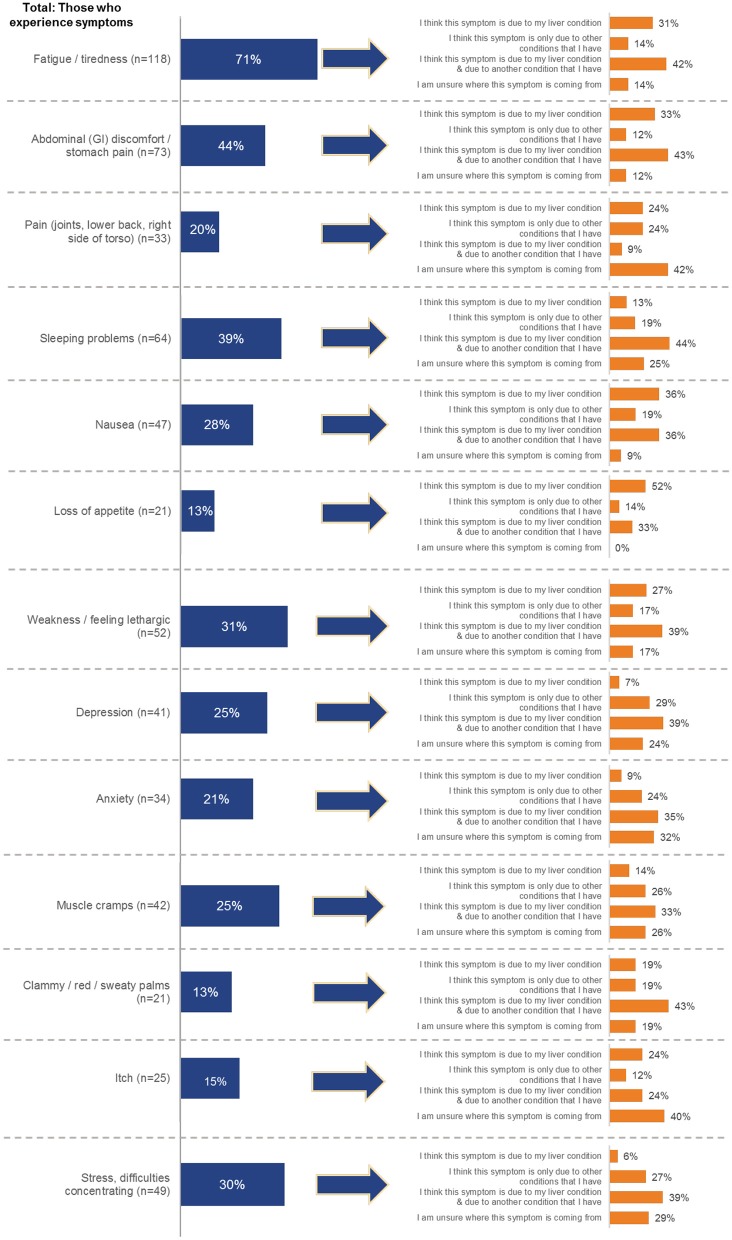
Overall summary of analysis of symptoms attributed to NASH. Patients were asked to indicate which symptoms they are experiencing, and then asked to indicate what was causing their symptoms.

When discussing symptoms, the most common reported symptom, not attributed to their liver condition, was fatigue/tiredness in 71% of cases [*n* = 118], followed by being obese/overweight in 62% of cases [*n* = 103] and abdominal pain in 44% of cases [*n* = 73]. Other symptoms were mentioned less frequently (Figure 4). Some patients were unable to differentiate whether certain symptoms they were experiencing originated from NASH or the comorbid condition (Figure 5). When asked about fatigue, 14% [*n* = 16] reported that they were unsure which of their health conditions contributed to this symptom and a further 14% did not associate fatigue as a symptom of their liver condition.

#### Evaluation of Potential New Products and Product Attributes

**Figure 6:**
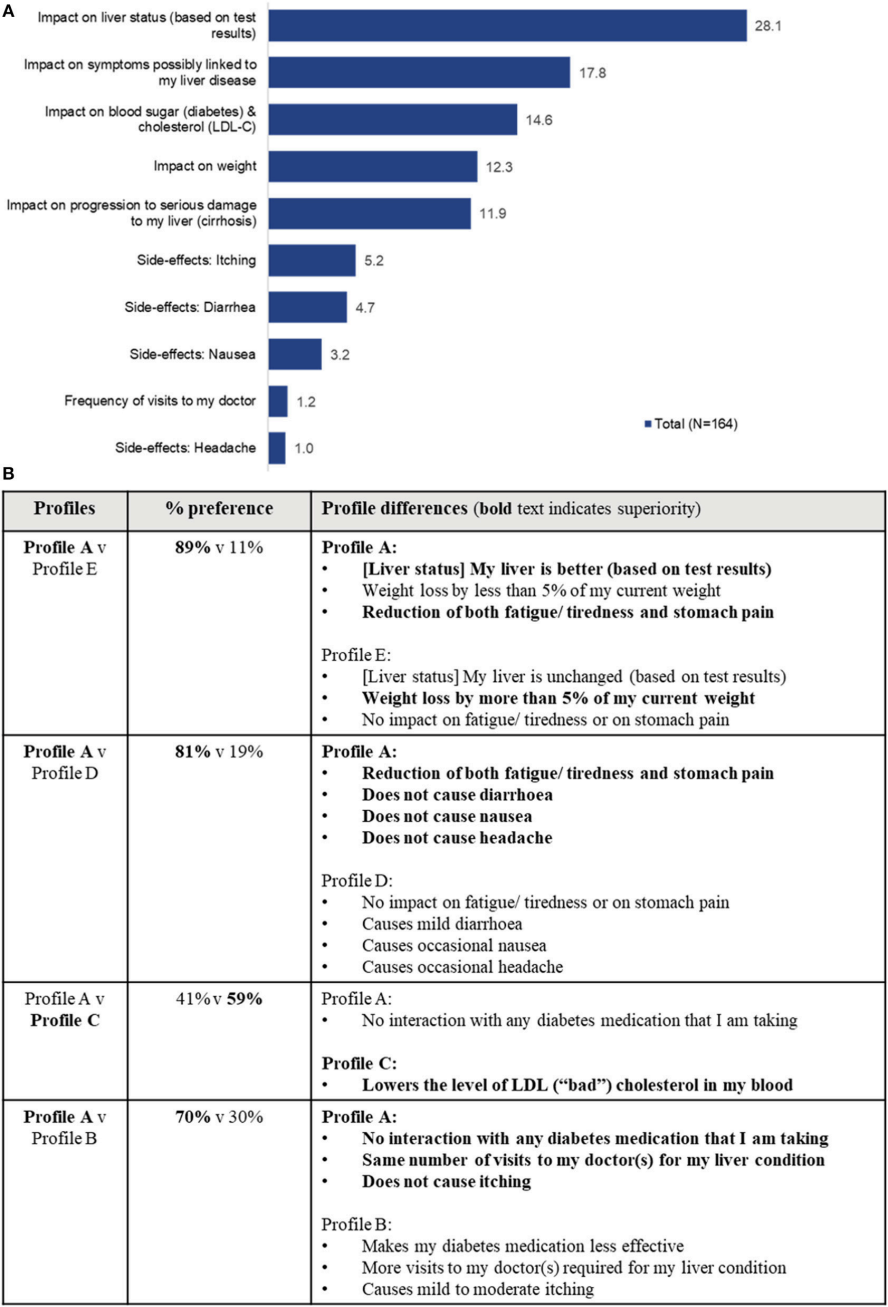
**(A)** Importance scores of a hypothetical product attributes as seen by patients. The total importance score is out of 100, if an attribute of a profile receives a score of 50 that means that half of all the importance of a profile is allocated to this attribute. **(B)** Summary of simulated patient preference based on the patient ACBC exercise responses.

Among attributes of a potential product profile during the ACBC exercise, “Impact on liver status (based on test results)” was ranked as the most important attribute overall, followed by “Impact on symptoms possibly linked to my liver disease” (Table 5, Figure 6). The other elements of the potential product profile were ranked lower, with side-effects and “Frequency of visits to my doctor” being the lowest ranked (least important) elements. Two out of the 166 patients were unable to assess the importance of the attributes.

**Table 5:** Relative importance as a percentage of different elements of a hypothetical product profile for patients. *(overall rank in brackets)*.

**Item**	**Label**	**Total (*N* = 164)**	**USA (*n* = 50)**	**Canada (*n* = 36)**	**Germany (*n* = 48)**	**UK (*n* = 30)**
**RELATIVE IMPORTANCE [%] (OVERALL RANK)**						
1	Impact on liver status (based on test results)	28.1 (1)	39.6 (1)	29.3 (1)	24.9 (1)	12.9 (4)
2	Impact on weight	12.3 (4)	21.5 (2)	4.7 (6)	10.0 (5)	9.8 (5)
3	Impact on symptoms possibly linked to my liver disease	17.8 (2)	16.8 (3)	23.1 (2)	16.0 (2)	15.9 (2)
4	Impact on blood sugar (diabetes) & cholesterol (LDL-C)	14.6 (3)	11.6 (4)	15.3 (3)	11.5 (4)	23.9 (1)
5	Frequency of visits to my doctor	1.2 (9)	2.6 (6)	0.0 (10)	0.2 (10)	1.7 (10)
6	Impact on progression to serious damage to my liver (cirrhosis)	11.9 (5)	5.1 (5)	13.6 (4)	15.7 (3)	15.3 (3)
7	Side-effects: Diarrhea	4.7 (7)	0.5	6.9 (5)	6.7 (7)	5.6 (7)
8	Side-effects: Nausea	3.2 (8)	0.3 (10)	2.4 (8)	6.1 (8)	4.2 (8)
9	Side-effects: Headache	1.0 (10)	0.5 (8)	1.1 (9)	0.4 (9)	2.8 (9)
10	Side-effects: Itching	5.2 (6)	1.5 (7)	3.5 (7)	8.6 (6)	7.9 (6)

A simulation was run, based on the patient ACBC exercise responses. Five potential product profiles (Table 6) were evaluated as part of the exercise. The results showed that patients had a preference of 59% for product profile C (Figure 6), a profile which lowered the low-density lipoprotein (LDL), in addition to providing efficacy on liver status and progression, and a minimal side-effect profile.

**Table 6:**
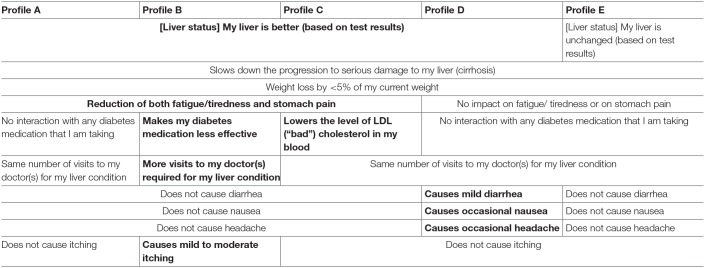
Overall characteristics of tested hypothetical products and differences (bolded), based on the patient ACBC exercise responses.

*Bold text indicates a differentiator used in the profiles (positive or negative)*.

#### HRQoL Results

**Figure 7:**
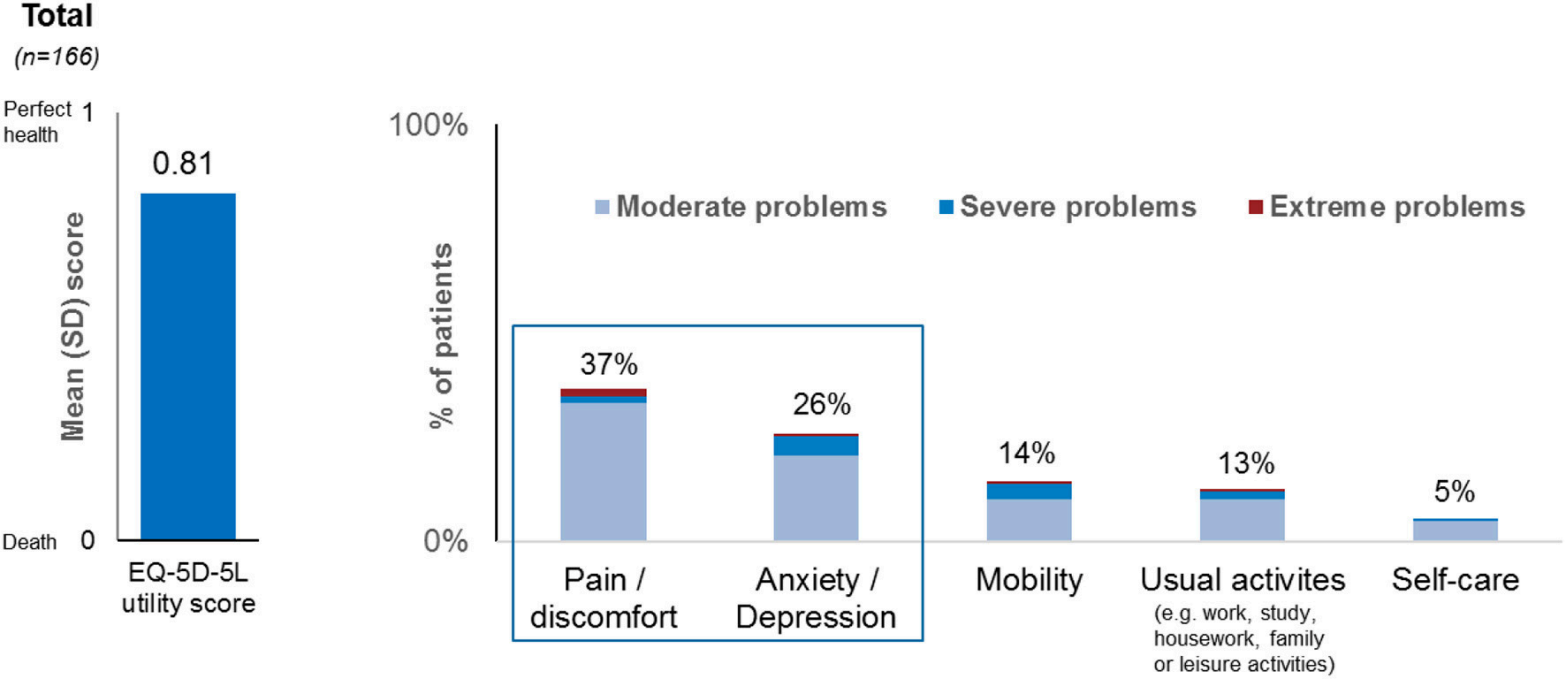
Overall summary of EQ-5D data across all countries.

The overall mean (SD) EQ-5D-5L utility score was 0.81 (0.17) across all patients. The most affected domains were pain/discomfort and anxiety depression for which 37% [*n* = 61] and 26% [*n* = 43] of respondents, respectively, reported moderate-extreme problems (Figure 7), with the remaining patients reporting “no problems” or “slight” problems. By contrast the impact was reported to be low on self-care, mobility and day to day working activities. The mean (SD) VAS was 67.2 (18.91).

#### Socio-Demographic Data

Overall, 64.4% [*n* = 107] of all respondents had employment full or part time or were self-employed (Table 3). 8.4% of respondents [*n* = 14] preferred not to disclose their employment status. The remaining respondents were out of work, unable to work, retired persons, home owners, students or military (Table 2).

#### Survey Satisfaction

**Figure 8:**
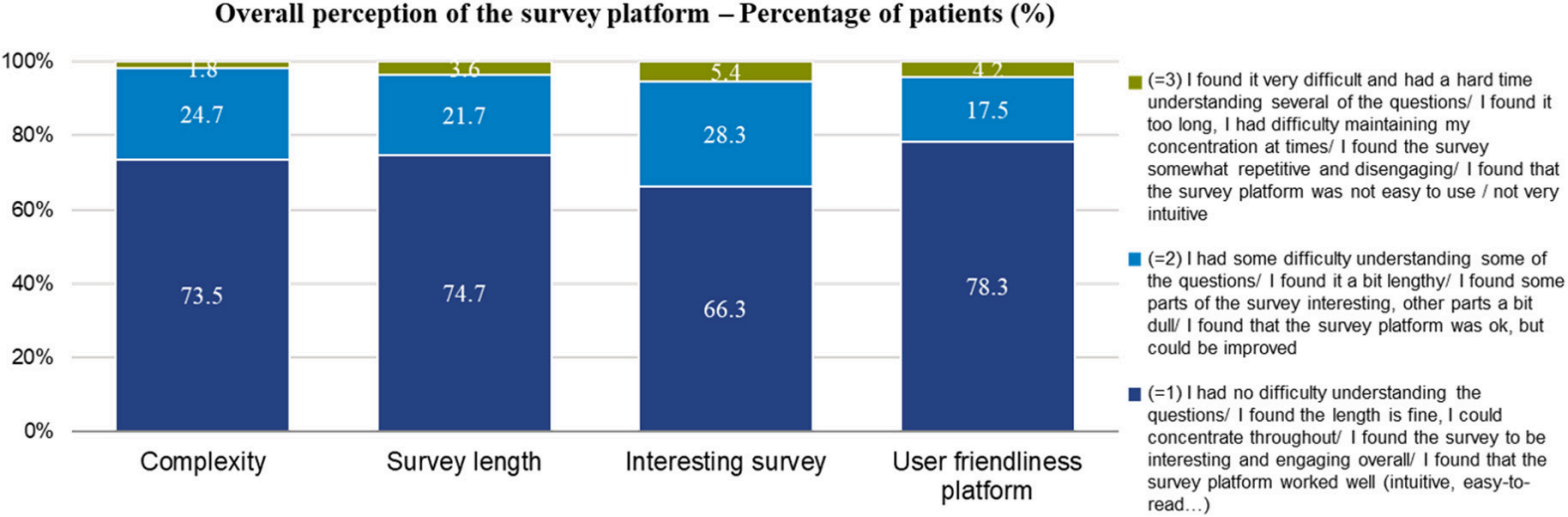
Overall results of survey experience across all four countries.

73.5% [*n* = 122] of respondents had no difficulty understanding the questions, 74.7% [*n* = 124] saw the survey length as being acceptable, 66.3% [*n* = 110] thought the survey questions were interesting, and 78.3% [*n* = 130] of respondents thought the survey platform worked well (Figure 8).

## Discussion

### The Design Phase

In addition to highlighting the experiences and the way in which patients with the diagnosis of NASH communicate about the disease, the results of the OBB helped to select the most important attributes for testing with patients. However, the resulting number of characteristics (attributes) to be tested exceeded what could be reasonably measured in a typical discrete choice experiment (DCE) trade-off design. Therefore, a combination of methodologies was employed, a ranking exercise, followed by a person's individual choice selection using the ACBC methodology.

The ACBC methodology combines aspects of Choice-Based Conjoint (CBC) and Adaptive Conjoint Analysis (ACA). Compared with other conjoint techniques, an ACBC interview is an interactive experience, customized to the preferences, and opinions of each respondent. The design is more stimulating and engaging, thus providing reliable results and a better experience for the patients (18). Another important learning from the OBB and the telephone interviews with patients was that patients have a limited knowledge of the disease and have difficulties in assigning specific symptoms to it. This information emphasized the need for formulating the questions very precisely in a language familiar to patients and to test the survey experience of the patients.

### Execution Phase

#### Patient Characteristics

There was equal gender distribution between males and females. The fibrosis staging was weighted more toward F2 rather than F3, indicating that the majority of patients were in the moderately advanced stage of their disease. Only 57% of patients reported being confirmed to have NASH via a liver biopsy. While this may be a reflection of the real-world practice, we cannot be certain that all NASH diagnoses would be confirmed if a biopsy is performed.

A high level of comorbidities was reported by the participants including obesity, diabetes or pre-diabetes, which is consistent with the published literature, in particular in the group of patients with advanced, non-cirrhotic NASH (19–21). Nevertheless, the comorbidities seen in the respondents appeared to be consistent to those of NASH patients.

#### NASH Patient Diagnosis

The vast majority of patients reported that their diagnosis was incidental, which is unsurprising, as liver conditions typically produce non-specific symptoms and often rely on histology to confirm the diagnosis, and NASH is no different in that sense (22).

Responses regarding the method by which patients received their diagnosis illustrated that non-invasive tools such as Ultrasound and transient elastography (FibroScan) are used frequently by physicians when NASH is suspected. As there were 43% of patients (72 out of 166) who did not report having their NASH confirmed via a liver biopsy (as per the clinical guidelines), it appears that there is a significant discrepancy between the clinical guidelines and real-world practice. The reasons for this discrepancy were not investigated in this study, however should be researched further. In the OBB, which had been conducted in the US and the UK, also only half of the patients reported a diagnostic confirmation by biopsy. When asked why biopsy had not been done, several patients conceded, that they were afraid of the pain associated with biopsy and therefore, avoided the procedure (16).

#### Information Satisfaction Levels and Symptomatology

Patient satisfaction with the information regarding NASH provided by their physician upon diagnosis appears to be quite high with 40% of patients indicating the highest satisfaction levels. However, this was not reflected in the patients' knowledge related to the disease, symptoms, and progression. On several occasions, patients who reported the symptoms they were experiencing struggled to assign them to NASH or any other condition they were suffering from. This highlights the need for patient education on these aspects of disease burden, to empower patients to understand, and manage their condition more effectively.

The high level of satisfaction reported in this study is also somewhat in contrast to some of the patients' statements in the preceding OBB, which indicated a rather high level of dissatisfaction with the information provided by the doctors at diagnosis (16). More in-depth research is warranted to identify and address the informational bottlenecks.

#### Evaluation of Potential New Product Attributes

The ranking exercise illustrated that patients place highest emphasis on aspects of a treatment related to impact on liver status and on symptoms possibly linked to their liver disease; an impact of a treatment on progression to serious damage to the liver (cirrhosis) was ranked lower, probably a reflection that patients are mostly unfamiliar with the progressive nature of their NASH and the meaning of cirrhosis. The (comparatively mild) side-effects tested in this research and visits to the doctor appeared to be of lower importance to the patients. The impact on blood sugar and cholesterol, or on weight were seen as having a high importance (ranked 3rd and 4th, respectively), which was consistent with the baseline patient characteristics, as many patients either had diabetes and/or were obese.

During the simulation, based on the ACBC exercise, potential new product profiles with superior “Impact on liver status,” had better outcomes. This implies that there is a 2-stage decision-making process taking place. First and foremost, the patient sees “Impact on liver status” as a pre-requisite for a patient to make a product choice. It appears that only if “Impact on liver status” is identical between potential new products the other features (e.g., lower LDL, as per product profile C) may become relevant to a patient. In this regard, weight loss >5% alone was not sufficient to compensate for a lack of impact on liver status (Profile E).

#### EQ-5D-5L Questionnaire

The overall utility score produced through the EQ-5D-5L questionnaire is of importance for health economists working in NASH. However, being a generic instrument the EQ-5D-5L designed to assess health status across diseases, might not be sensitive enough to capture specific impairment due to NASH. These results are, nonetheless, valuable results as they also allow the researchers to compare scores generated from NASH patients with other conditions. Valuable insights which seem to confirm other published research (12, 23) show higher impairment on “pain/discomfort” and “anxiety/depression.” In order to better characterize HRQoL impairment in NASH, validated, disease-specific tools could be used in addition to generic tools.

#### Survey Satisfaction

The overall survey satisfaction was high, as only a small minority of patients reported having challenges with the survey. This indicated that the cognitive burden of the survey was low, thereby increasing the likelihood of accurate and meaningful responses. The high survey satisfaction also validates the decision to use the ranking exercise together with ACBC methodology, as this ensured that a sufficient number of questions was asked to achieve the survey outputs, while not putting an excessive burden on the respondents.

### Patient Preference Study Utility

Patient preference studies such as this one, could help inform HTA and regulatory authorities on what features of new treatments patients would value and what trade-offs they are willing to make. These trade-offs are impacted by several factors, among them the disease particularities, and therefore they should be elicited in well-designed studies with the right target patient population. Data from such studies can help inform on acceptability of medication profiles to the patients and contribute to the drug development process, regulatory and access decisions (24). In this research we saw that patients were uncompromising on “Impact on liver status” and appreciated the lower LDL levels if liver efficacy was attained. There are currently no therapies available for NASH patients that would treat their condition or alleviate their symptoms. In the future, however, when faced with multiple efficacious therapies, patient choice may be different as the patients would know that they are facing a real choice with regards to their therapy, rather than a hypothetical exercise. The application of patient preference studies is growing, as exemplified by the multi-stakeholder collaborative initiative IMI PREFER (25). Our study with NASH patients is a contribution to this field with a special emphasis on describing patient preferences with the objective of supporting product development and identification of outcomes that matter most to patients.

### Study Strengths

This study invited patients to share their preferences relating to a number of hypothetical product profiles and their elements, such as efficacy and side-effects, which could inform NASH stakeholders in the future and focus clinical research to achieve better patient outcomes. A strength of the study design was the ability to test a large number of hypothetical product attributes by using a combination of a ranking exercises and the ACBC methodology. The use of a step-by-step design phase, including the survey testing with patients, helped to create a lean, and focused quantitative survey phase. Furthermore, the low cognitive burden of the survey meant we had a high completion rate and increased the likelihood of this research presenting objective, good quality, accurate, and meaningful patient responses that reflect the current experiences of patient with the diagnosis of NASH with fibrosis staging F2 or F3.

### Study Limitations

The study sample may have included NAFLD patients, because not all patients were confirmed to have NASH with a liver biopsy. The results of this study are only relevant for the patients who fulfilled the inclusion criteria for this study, which excluded F1 and F4 patients, as determined by transient elastography or liver biopsy. Therefore, the study population may not be representative of the entire NASH patient population in general, albeit it is reasonably representative of those patients seen and managed by hepatologists in clinical practice. The level of patient knowledge often differs by the recruitment setting; for this research, the majority of patients were recruited through hepatology centers via physician referrals, which could have introduced sample bias, as these patients are not being managed by primary care physicians, who may be managing diagnosed NASH patients in some countries (N.B.: in countries like Germany, NASH patients are typically not managed for their liver disease at the primary care physician level). The consistency of the study results with the decisions in the real-world setting is yet to be confirmed, as patients may act differently when presented with the choice in real life. Statistical significance testing was not conducted as part of the research due to a relatively small sample.

This research was not geared toward investigating subgroup heterogeneity nor to highlight cross-country comparisons. Clearly defined subgroups with large sample sizes would be required in order to make a robust comparison with any degree of statistical significance. While this research does provide country-level data in the figures and tables, these have not undergone statistical significance testing. Country-level data was also not called out in the results or the discussion, as any derivation would not be accurate due to the effects from cultural differences and respondent variation, which was not adjusted for in this research due to a relatively small country-level sample size.

## Conclusions

“Impact on liver status” is the primary outcome sought by NASH patients, while other outcomes appeared to be secondary for the patients in this study. Patients demonstrated a general lack of understanding of the disease and did not seem familiar with consequences of NASH in the long-term. The limited communication by physicians regarding NASH-targeted interventions, e.g., weight loss or exercise, reflects the current nihilism in the standard of care, however, to a patient this may be interpreted that NASH is not such a serious condition. There is an assumption that if patients were to perceive NASH more seriously and understand the emerging severe long-term consequences, this could be a driver of better disease management and improved patient outcomes. This is because with improved understanding it is assumed that patients may start engaging in behaviors which would address some of the comorbidities and severe long-term consequences of NASH. If this hypothesis is true, it could mean that biopsied patients have better long-term outcomes due to a more in-depth communication with their treating physician and hence an improved understanding of NASH. However, further research would be required comparing those patients who are biopsied with non-biopsied patients to support this hypothesis. It is therefore necessary to improve communication of NASH consequences and prognosis in order to improve patient understanding of their disease and the need for a confirmatory diagnosis and disease monitoring. In parallel, patients would require support systems to help them make positive changes to their lifestyles. Additional research on NASH patient preferences is also needed to better appreciate patient needs across the broader NASH population to inform stakeholders involved in development of new therapies for NASH and delivery of care.

## Data Availability

All datasets generated for this study are included in the manuscript and/or the Supplementary Files.

## Author Contributions

NC and M-MB drove the study, shaped the design of the study, and this publication. All authors took part in overseeing this study, contributing their ideas on an ongoing basis, and reviewed this paper.

### Conflict of Interest Statement

NC and M-MB are employees of Novartis Pharma AG. AG sits on steering committees for: Gilead, Intercept, Novartis; Advisor: AbbVie, Alexion, BMS, Gilead, Intercept, Ipsen, Novartis, Pfizer, Sequana; Speaker for: AbbVie, Alexion, BMS, CSL Behring, Falk, Gilead, Intercept, Novartis, Sequana; Provides Research Support for: Intercept (NAFLD CSG), Novartis, Kibion, Exalenz (LITMUS). GH has provided consultancy services for Gilead, Intercept, Novartis and Cymabay. JS reports consultancies with AbbVie, BBN Cardio, Intercept Pharmaceuticals, Galmed, Genfit, Gilead Sciences, IQVIA, Medimmune, Novartis, Pfizer; research funding from Gilead Sciences, Yakult Europe B.V.; travel support: Janssen; lectures for Falk Foundation, Takeda, Merck, Norgine. AS sits on a steering committee for Novartis and delivered a presentation for Lilly. The remaining author declares that the research was conducted in the absence of any commercial or financial relationships that could be construed as a potential conflict of interest.
